# A cutting-edge deep learning-and-radiomics-based ultrasound nomogram for precise prediction of axillary lymph node metastasis in breast cancer patients ≥ 75 years

**DOI:** 10.3389/fendo.2024.1323452

**Published:** 2024-07-12

**Authors:** Lang Qian, Xihui Liu, Shichong Zhou, Wenxiang Zhi, Kai Zhang, Haoqiu Li, Jiawei Li, Cai Chang

**Affiliations:** ^1^ Department of Medical Ultrasound, Fudan University Shanghai Cancer Center, Shanghai, China; ^2^ Department of Oncology, Shanghai Medical College, Fudan University, Shanghai, China; ^3^ School of Optical-Electrical and Computer Engineering, University of Shanghai for Science and Technology, Shanghai, China

**Keywords:** deep learning, radiomics, elderly women, axillary lymph node metastasis, ultrasound

## Abstract

**Objective:**

The objective of this study was to develop a deep learning-and-radiomics-based ultrasound nomogram for the evaluation of axillary lymph node (ALN) metastasis risk in breast cancer patients ≥ 75 years.

**Methods:**

The study enrolled breast cancer patients ≥ 75 years who underwent either sentinel lymph node biopsy or ALN dissection at Fudan University Shanghai Cancer Center. DenseNet-201 was employed as the base model, and it was trained using the Adam optimizer and cross-entropy loss function to extract deep learning (DL) features from ultrasound images. Additionally, radiomics features were extracted from ultrasound images utilizing the Pyradiomics tool, and a Rad-Score (RS) was calculated employing the Lasso regression algorithm. A stepwise multivariable logistic regression analysis was conducted in the training set to establish a prediction model for lymph node metastasis, which was subsequently validated in the validation set. Evaluation metrics included area under the curve (AUC), accuracy, sensitivity, specificity, positive predictive value, negative predictive value, and F1-score. The calibration of the model’s performance and its clinical prediction accuracy were assessed using calibration curves and decision curves respectively. Furthermore, integrated discrimination improvement and net reclassification improvement were utilized to quantify enhancements in RS.

**Results:**

Histological grade, axillary ultrasound, and RS were identified as independent risk factors for predicting lymph node metastasis. The integration of the RS into the clinical prediction model significantly improved its predictive performance, with an AUC of 0.937 in the training set, surpassing both the clinical model and the RS model alone. In the validation set, the integrated model also outperformed other models with AUCs of 0.906, 0.744, and 0.890 for the integrated model, clinical model, and RS model respectively. Experimental results demonstrated that this study’s integrated prediction model could enhance both accuracy and generalizability.

**Conclusion:**

The DL and radiomics-based model exhibited remarkable accuracy and reliability in predicting ALN status among breast cancer patients ≥ 75 years, thereby contributing to the enhancement of personalized treatment strategies’ efficacy and improvement of patients’ quality of life.

## Introduction

In recent years, the incidence of breast cancer has been steadily increasing, which is closely associated with the application and popularization of imaging diagnostic techniques. According to data from the Chinese Cancer Registry Annual Report, the proportion of elderly breast cancer patients in China was approximately 16.5% in 2008 and is projected to increase to 27% by 2030 (1). This suggests that there is a continuous upward trend in the incidence of breast cancer among elderly women.

The lymph node metastasis (LNM) status of breast cancer patients plays a pivotal role in determining disease staging and predicting prognosis. The sentinel lymph node (SLN), which receives the initial drainage from the breast cancer, holds significant importance in predicting the axillary lymph nodes (ALN) status (2). This information is crucial for establishing breast cancer staging, formulating treatment plans, and predicting prognosis. Although SLN biopsy (SLNB) is the standard axillary staging method, it has limitations including complications such as arm numbness or lymphedema. While the false-negative rate is acceptable, there are still cases (7.8–27.3%) that may have been missed for LNM (3–6). Adjuvant therapies like chemotherapy and radiotherapy can reduce the impact of false negative SLNB on prognosis for breast cancer patients of other ages, but not all elderly patients can receive postoperative adjuvant treatment due to their complex health status and many complications. These limitations significantly impact the treatment and prognosis of elderly women with breast cancer. Elderly women often exhibit relatively favorable biological features including hormone receptor status, tumor grade, and proliferation rate (7). However, due to their complex health conditions and presence of multiple complications, treating elderly patients with breast cancer can be more challenging (8). Existing evidence is relatively limited, making it difficult to develop personalized treatment plans (9–11). The International Society of Geriatric Oncology and European Breast Cancer Experts have proposed new recommendations suggesting that ALN surgery may be spared in clinically negative elderly patients without axillary nodes involvement (12). Therefore, accurate preoperative assessment of ALN metastasis becomes particularly significant for avoiding unnecessary surgeries and developing personalized treatment plans.

Ultrasound is a widely employed technique for preoperative assessment of ALN due to its simplicity, high-resolution soft tissue imaging capabilities, and absence of radiation exposure. However, it has limitations in accurately diagnosing ALN metastasis, with an area under the curve (AUC) ranging from 0.585 to 0.719, indicating restricted diagnostic ability (13). To overcome this limitation, recent research has shifted focus towards the primary breast lesion with the aim of predicting ALN metastasis by analyzing imaging features and clinicopathological factors associated with the primary tumor. Several studies have demonstrated that clinical and imaging characteristics such as tumor size, histological grade, and patient age possess significant predictive potential for LNM. Furthermore, prediction models based on these features have exhibited robust performance in prognostication (14–16). Nevertheless, previous studies often lacked representation of elderly patients while conventional clinical prediction models had limitations such as subjective interpretation influenced by individual expertise or insufficiently detailed description of image analysis features. Future research should prioritize inclusion of elderly breast cancer patients while enhancing and optimizing imaging analysis methods to improve prediction model accuracy and clinical feasibility.

However, new opportunities have emerged in the field of medical imaging with the rapid development of radiomics and deep learning (DL). Radiomics and DL have tremendous potential for application in the diagnosis of breast cancer. Traditional radiomics offers several advantages, including high reliability, strong interpretability, and minimal data requirements. On the other hand, DL possesses distinct strengths such as automatic feature learning, effective representation of complex structures, powerful expression ability for features, and enhanced accuracy (17, 18). By integrating these two techniques, the respective strengths of each can be effectively harnessed to augment the precision and dependability of breast cancer diagnostic models. For instance, the integration of radiomics and DL techniques enables the identification of characteristic textures and details that are imperceptible to the human eye, facilitating quantitative and objective evaluations of tumor properties.

The objective of this study was to establish a predictive model utilizing DL and radiomics techniques for the assessment of ALN metastasis risk in breast cancer patients aged ≥75 years. We would collect imaging data and clinicopathological information from breast cancer patients, followed by image analysis and feature extraction using advanced DL and radiomics techniques. Through comparison and validation against actual LNM status, we aimed to evaluate the accuracy and reliability of our model, enabling surgeons to make more precise and evidence-based clinical decisions.

## Methods

### Patients

This retrospective study included cases from pathology examinations of breast cancer surgeries conducted at Shanghai Cancer Center, Fudan University, between January 2018 and June 2021. The study population comprised patients aged ≥75 years who underwent either SLNB or ALN dissection (ALND). Cases were excluded if they had incomplete clinical or pathological data, received prior neoadjuvant therapy, or presented with other malignancies. This study was approved by the Ethics Committee of Shanghai Cancer Institute, Fudan University. As this study is retrospective in nature and does not disclose any identifiable information, informed consent was deemed unnecessary.

### Clinicopathologic feature

This study comprehensively collected and analyzed various clinicopathological features, including age, age at menopause (defined as the year of the last menstrual period), reproductive age (defined as the age at first childbirth), body mass index (BMI, calculated based on height and weight), family history, tumor palpability, tumor location, tumor diameter, anteroposterior diameter, Breast Imaging Reporting and Data System (BI-RADS) level, height-to-width ratio, multifocality, histologic grade, histologic type, estrogen receptor (ER) status, progesterone receptor (PR) status, human epidermal growth factor receptor 2 (HER-2) overexpression, Ki-67 expression, molecular subtype and axillary ultrasound.

Immunohistochemistry staining was utilized to assess the expression of ER, PR, and Ki-67 in breast cancer tissues. Tumors demonstrating Ki-67 expression levels exceeding 14% were classified as having high expression. ER and PR positivity were defined as positive staining observed in at least 1% of tumor cells. HER-2 overexpression was evaluated following the guidelines established by the American Society of Clinical Oncology/American Pathology Association. Breast cancer molecular subtypes were categorized based on the consensus reached during the 2019 St. Gallen International Breast Cancer Conference, including Luminal A, Luminal B (comprising both HER-2 positive and HER-2 negative), HER-2 overexpression type, and triple-negative breast cancer type. Axillary ultrasound assessment was performed under ultrasound guidance using BI-RADS classification criteria. Abnormal lymph nodes detected via axillary ultrasound were considered suspicious if they exhibited at least one suspicious feature such as complete or partial substitution with unclear or irregular masses, complete or partial disappearance of lymph sinuses, focal or irregular cortical thickening, diffuse cortical thickening ≥3 mm, presence of microcalcifications within lymph nodes, or non-lymphatic portal blood flow signals on color doppler; otherwise, they were deemed negative.

To preserve the scientific validity of our study, we implemented a blinded assessment protocol for the radiographic analysis. Radiologists, uninformed about patients’ histopathological results, were restricted to the evaluation of images alone, with no access to medical records. An anonymized coding system was employed to label each case, ensuring that the correlation between radiological and pathological data was independently analyzed by a third-party investigator, who was not involved in imaging interpretation or clinical management. Finally, we maintained a strict separation between radiologic and pathologic datasets during the statistical analysis to reinforce the objectivity and accuracy of our assessment.

### Deep learning features

Using the graphical image annotation tool Labelme, we manually selected a rectangular region of interest (ROI) on the ultrasound image representing the maximum cross-section of the tumor, encompassing the entire tumor area, including both the complete hypoechoic tumor region and any present echogenic halo, as well as additional hypoechoic tumor regions (19).

The grayscale ultrasound images were processed using DenseNet-201 as the base model, and its generalization performance was enhanced by incorporating pre-trained weights from ImageNet (20, 21). To align with the standardized weights of the ImageNet dataset, we expanded the original single-channel grayscale ultrasound image into a three-channel image through contrast enhancement based on histogram equalization and denoising based on wavelet transform.

In the network training phase, we employed Adam as the optimizer and selected cross-entropy as the loss function (22). To enhance model convergence speed and mitigate overfitting tendencies, we implemented a step-based learning rate decay strategy and an early stopping training strategy (23). Specifically, we initialized the learning rate to 0.001 and reduced it by half every 50 epochs. Training was terminated if there was no improvement in model performance for more than 50 epochs. Additionally, to facilitate efficient utilization of computing resources and memory consumption, we set the batch size to 8.

**Figure 1:**
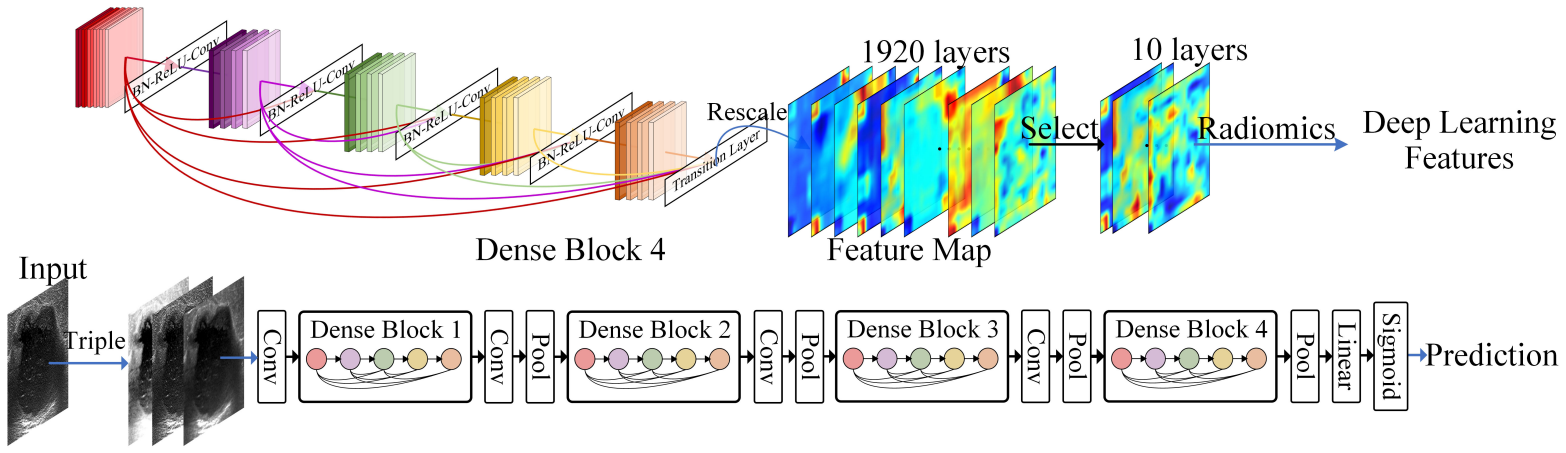
Deep learning feature extraction. The 1920 feature maps of the last densely connected convolutional layer are scaled to the input size, and 10 feature maps are selected to extract deep learning features using Radiomics.

The following methods were employed in this study to effectively extract DL features. Firstly, during the forward propagation of the network, we rescaled the feature maps to match the original input size and extracted radiomics features by utilizing manually delineated ROI regions. Considering that DenseNet-201 consists of 1920 nodes in its last layer, for enhanced computational efficiency and reduced redundancy, we replaced the softmax activation function in the final layer with sigmoid and approximated the binary classification task as a prediction task. Next, we sorted the weights corresponding to these 1920 nodes based on their absolute values and selected a subset of “key nodes”. We then filtered these key nodes using Spearman correlation coefficient by calculating correlations between nodes on the training set and removing those unrelated to key nodes. Finally, we selected 128 nodes from which radiomics features were extracted from their respective feature maps. It should be noted that while the feature maps of the network can reflect characteristics of ultrasound images, they cannot fully represent them. Therefore, only first-order features were extracted from these feature maps in this study. The process of DL feature extraction is shown in **Figure 1**.

### Radiomics features and Rad-Score calculation

We utilized the Labelme tool for manual annotation of ROI on maximum cross-sectional ultrasound images of tumors, tracing along the contours of the tumors. The manual segmentations were performed by two radiologists who were blinded to the pathological results. Each manual segmentation was repeated twice to assess inter- and intra-observer reproducibility (24). Subsequently, we employed the Pyradiomics tool (25) to extract features from these ROI regions. These features encompassed 480 radiomics features, shape features, histogram features and texture features. All feature extraction algorithms adhered to the standards proposed by the Image Biomarker Standardization Initiative (ISBI). Next, we validated the stability of these radiomics features using intra-/inter-class correlation coefficients (ICCs), retaining only those with an ICC greater than 0.6. Furthermore, we applied the minimum redundancy and maximum relevance (mRMR) (26) algorithm to select relevant features from both retained radiomics and DL ones, resulting in a final selection of 32 features. Finally, we employed Lasso regression algorithm to calculate Rad-Score (RS).

### Nomogram construction

**Figure 2:**
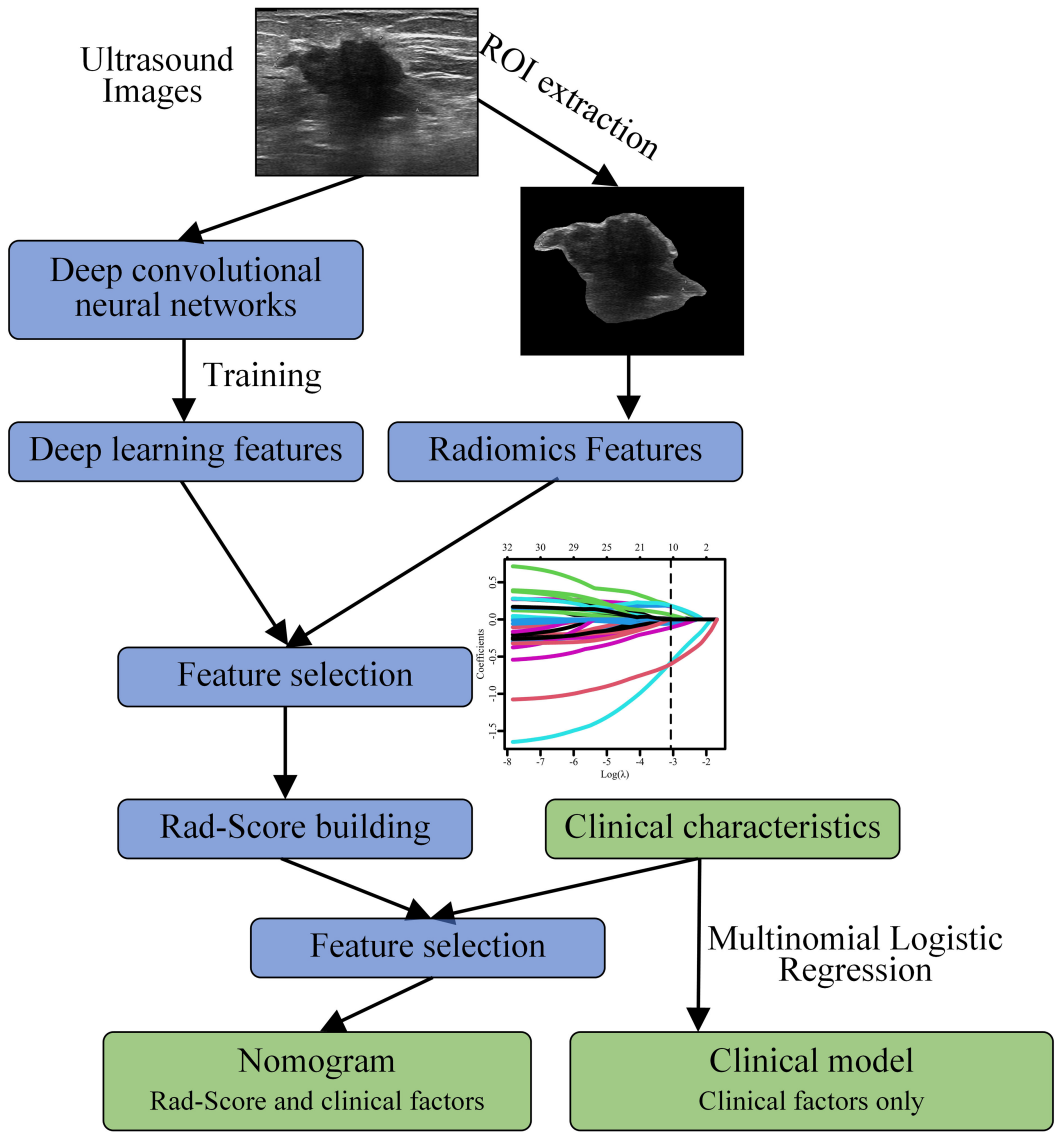
Workflow for constructing a nomogram model based on deep learning and radiomics features to predict the risk of axillary lymph node (ALN) metastasis in breast cancer patients ≥ 75 years.

**Figure 2** shows the workflow of this study. The data processing in this study was conducted using SPSS 25.0 and R 4.2.2. Continuous data were presented as mean ± standard deviation, while categorical data were presented as frequencies. Univariate analysis was performed to identify variables (including RS values) associated with LNM. Fisher’s exact test or chi-square test was used for between-group comparisons of categorical variables, and the t-test or Mann Whitney U test was used for continuous variables. In the training set, a stepwise multivariate logistic regression approach was employed to establish a predictive model for LNM. Variables with a P < 0.05 in the univariate analysis were included in the logistic regression analysis, and those with a P < 0.05 in the multivariate analysis were included in the final predictive model. The performance of the model was validated using the validation set. To achieve better generalizability, we utilized Youden index to determine the final threshold (cut-off value) (27).

### Evaluation index

In this study, the performance and predictive accuracy of the model were evaluated using AUC, accuracy, sensitivity, specificity, positive predictive value (PPV), negative predictive value (NPV), and F1-score. The calibration curve and decision curve were used to assess the calibration and clinical prediction accuracy of the model (28). The added value of RS values to the model performance was evaluated using the integrated discrimination improvement (IDI) and net reclassification improvement (NRI) indices (29).

## Results

### Baseline characteristics

The study included 364 elderly breast cancer patients aged ≥75 years, with a median age of 77 years. All patients underwent either breast-conserving surgery or mastectomy on the ipsilateral side, accompanied by SLNB or ALND following a pathological diagnosis of breast cancer. Among these patients, 281 underwent SLNB, and 106 patients underwent ALND. Based on the pathological findings, LNM was detected in 118 patients (32.4%), while no evidence of LNM was observed in 246 patients (67.6%).

The clinical factors are presented in **Table 1**. To ensure comparable proportions of positive and negative patients, a stratified sampling approach was employed to group the training set and validation set based on time (93/196 in the training set, 25/50 in the validation set). No significant differences were observed in these clinical features or LNM status between the training and validation sets (P > 0.05). Univariate analysis of indicators between positive and negative cases of ALN metastasis revealed significant differences in certain features, including location of the mass, histological type, and axillary ultrasound etc.

**Table 1:** Patients’ characteristics and univariate analysis of variables for lymph node metastasis.

Variables	Training set (n=289)			Validation set (n=75)		
	ALN-	ALN+	P	ALN-	ALN+	P
**Age(y)**	78 ± 3	79 ± 4	0.063	78 ± 3	79 ± 3	0.684
**Menopausal age(y)**	50 ± 3	50 ± 4	0.188	51 ± 3	50 ± 4	0.086
**Reproductive age(y)**	26 ± 4	25 ± 4	0.019	25 ± 4	25 ± 3	0.775
**BMI(kg/m^2^)**	23.9 ± 3.5	24.1 ± 3.6	0.683	23.2 ± 3.2	24.6 ± 3.0	0.062
**Anteroposterior diameter (mm)**	14.0 ± 7.0	16.7 ± 7.4	0.004	14.2 ± 6.3	16.7 ± 8.5	0.159
**Maximum diameter(mm)**	21.9 ± 14.8	25.7 ± 14.7	0.042	23.5 ± 11.6	24.9 ± 9.9	0.622
**Family history**			0.976			0.15
No	162	77		46	20	
Yes	34	16		4	5	
**Palpability**			0.852			0.597
No	16	7		2	2	
Yes	180	86		48	23	
**Location of the mass**			0.001			0.022
Outer upper	74	56		21	10	
Lower outer	27	12		7	7	
Upper inner	53	8		16	5	
Lower inner	25	10		6	0	
Posterior to the nipple	17	7		0	3	
**Histological grade**			0.269			0.479
I-II	127	54		36	16	
III	69	39		14	9	
**Multifocality**			0.472			1
No	180	83		45	23	
Yes	16	10		5	2	
**Histological type**			<0.001			0.157
IDC	135	83		32	20	
Others	61	10		18	5	
**HER-2 expression**			0.117			0.286
Negative	170	74		45	20	
Positive	26	19		5	5	
**ER status**			0.889			0.221
Negative	47	23		8	7	
Positive	149	70		42	18	
**PR status**			0.271			0.866
Negative	65	37		19	9	
Positive	131	56		31	16	
**Ki-67 expression**			0.122			0.867
Low	84	31		19	10	
High	112	62		31	15	
**Molecular type**			0.267			0.574
LuminalA	78	28		18	8	
LuminalB (HER-2-)	62	35		20	7	
LuminalB (HER-2+)	9	8		4	3	
HER-2 enriched	17	11		1	2	
TNBC	30	11		7	5	
**Axillary US**			<0.001			<0.001
Negative	180	47		46	12	
Suspicious	16	46		4	13	
**BI-RADS US**			<0.001			0.003
3–4A	19	4		5	1	
4B or 4C	163	63		44	17	
5	14	26		1	7	
**Taller-than-wide shape**			0.173			0.657
<1	176	88		45	24	
≥1	20	5		5	1	
**RS**	-1.606 ± 1.43	0.213 ± 0.83	<0.001	-1.770 ± 1.37	0.296 ± 0.63	<0.001

ALN, axillary lymph node; BMI, body mass index; IDC, invasive ductal carcinoma; TNBC, triple-negative breast cancer; US, ultrasound; BI-RADS, Breast Imaging Reporting and Data System; RS, Rad-Score.

### Feature screening and Rad-Score calculation

The DenseNet201 model served as the foundational architecture in this study, with a prediction approach utilizing the sigmoid activation function to approximate the classification task. Subsequently, the last layer consisting of 1920 nodes was sorted based on their weights, and after analysis using Spearman’s correlation coefficient, only 9 nodes exhibiting a correlation coefficient below 0.1 were retained from the remaining nodes. This resulted in a total of 190 features derived from these selected nodes.

**Figure 3:**
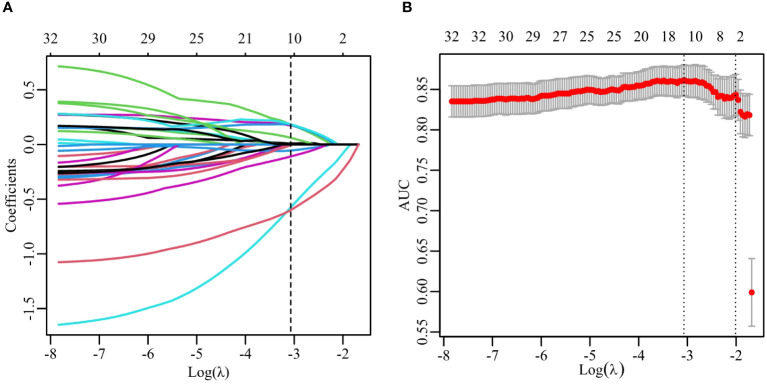
Feature selection with the LASSO algorithm. **(A)** LASSO coefficient with different λ. The dashed line represents the selection of 11 features using the optimal λ (0.046). **(B)** The selection of LASSO model’s parameter using 5-fold cross-validation. The dashed lines corresponds to two values which the smallest mean squared error (MSE) is achieved (0.046) and MSE is within one standard error of the smallest(0.135).

According to the aforementioned method, each image can extract a total of 670 features, which included 190 DL features and 480 radiomics features. Consequently, with two images per patient, there were a total of 1340 image features. After validation using ICCs coefficient and mRMR algorithm, only 32 features were retained. Further analysis utilizing the Lasso algorithm resulted in the selection of 20 image features for constructing the RS. These selected features consisted of 7 DL features and 4 radiomics features as presented in **Table 2**. The process of feature selection using the Lasso algorithm is illustrated in **Figure 3**.

**Table 2:** Radiomics signatures calculation formula.

	(Intercept)	-1.00838
**Deep learning features**	original_firstorder_Range_750_1	0.009664
	original_firstorder_RootMeanSquared_992_0	0.175046
	original_firstorder_StandardDeviation_273_1	0.18031
	original_firstorder_Mean_992_0	-0.0043
	original_firstorder_10Percentile_992_1	-0.05537
	original_firstorder_Maximum_750_0	0.060832
	original_firstorder_Minimum_1413_1	-0.10877
**Radiomics features**	original_glcm_Correlation.0	0.182627
	wavelet.LL_glcm_ClusterShade.1	0.184388
	wavelet.LH_firstorder_Mean.1	-0.57384
	wavelet.LH_firstorder_Mean.0	-0.59918

### Model construction and result analysis

Variables with P < 0.05 in the univariate analysis were included in the multivariate analysis. Regression models were constructed for both pure clinical factors and factors incorporating RS values. As depicted in **Table 3**, the multivariate analysis revealed significant disparities in tissue classification and lymph node description under both scenarios, while RS values also emerged as an important predictive factor within the model presented in this study.

**Table 3:** Variables and coefficients of the clinical model and integrated model

Factors	Clinical model			Factors	Integrated model		
	β	P	Adjusted OR		β	P	Adjusted OR
**Reproductive age**	-0.066	0.100	0.936	**Histological type**	-0.979	0.059	0.376
**Location of the mass**	-0.202	0.070	0.817	**Axillary US**	2.560	<0.001	12.931
**Histological type**	-0.981	0.014	0.375	**RS**	1.613	<0.001	5.018
**Axillary US**	2.198	<0.001	9.006				
**Constant**	1.990			**Constant**	0.790		

OR, Odds Ratio; US, ultrasound; RS, Rad-Score.

The study utilized RS values as a singular variable for predicting LNM and constructed a univariate regression model, which was compared to the multivariate model in order to further demonstrate the enhanced performance contributed by RS values in **Table 4** and **Table 5**. The proposed model demonstrated exceptional prediction accuracy and generalization performance, with an AUC of 0.906, representing improvements of 1.8% and 21.8% when compared to the RS model and pure clinical model respectively. By incorporating RS values into the clinical factors, the accuracy of the model increased from 0.733 to 0.827, indicating an improvement of 12.7%. Furthermore, the F1-score increased from 0.583 to 0.754, showing an improvement of 29.3% compared to the clinical model alone. Additionally, inclusion of RS values resulted in an NRI of 0.306 and an IDI of 0.320 when compared to using only pure clinical factors; this provides compelling evidence that utilizing RS values obtained through our proposed method enhances both predictive accuracy and generalization performance.

**Table 4:** Performance of the integrated model, RS model and clinical model on training set

Model	AUC	Accuracy	Sensitivity	Specificity	PPV	NPV	F1-score
Integrated model	0.937	0.844	0.882	0.827	0.707	0.936	0.785
RS model	0.905	0.782	0.860	0.745	0.615	0.918	0.717
Clinical model	0.784	0.782	0.634	0.852	0.670	0.831	0.652

AUC, area under the curve; RS, Rad-Score; PPV, positive predictive value; NPV, negative predictive value.

**Table 5:** Performance of the integrated model, RS model and clinical model on validation set.

Model	AUC	Accuracy	Sensitivity	Specificity	PPV	NPV	F1-score
Integrated model	0.906	0.827	0.800	0.840	0.714	0.894	0.755
RS model	0.890	0.787	0.800	0.780	0.645	0.886	0.714
Clinical model	0.744	0.733	0.560	0.820	0.609	0.788	0.583

AUC, area under the curve; RS, Rad-Score; PPV, positive predictive value; NPV, negative predictive value.

**Figure 4:**
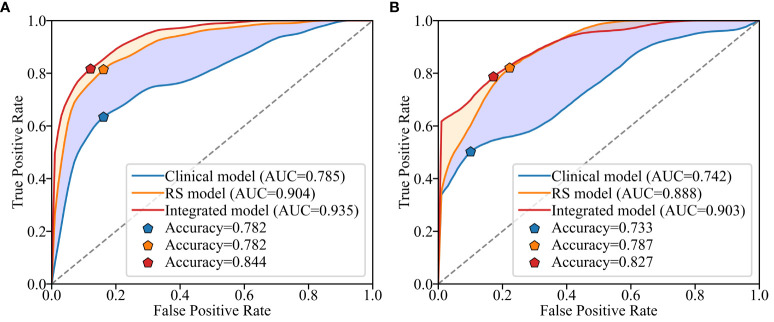
Receiver operating characteristic (ROC) curves for different models on the training set **(A)** and validation set **(B)**.

**Figure 5:**
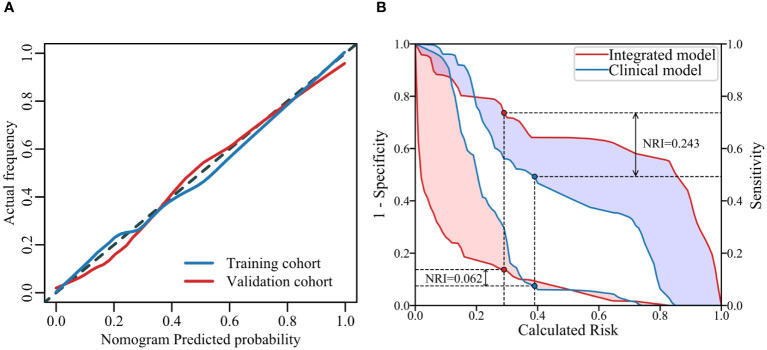
Calibration curves of the model on the training and validation sets **(A)** illustrates the agreement between the risk predicted by the nomogram and the true outcomes. A closer fit of the solid line to the dotted line indicates better predictive accuracy. The IDI curves of two models **(B)** shows the False Positive Rate (FPR) and True Positive Rate (TPR) at different thresholds, highlighting the differences between the models at their respective optimal threshold points.

The ROC curves of the three models on the validation set are depicted in **Figure 4**. It is evident that the Rad-Score itself demonstrates remarkable predictive accuracy, and when combined with clinical factors, the model achieves its highest prediction accuracy. In this study, we employed the Youden Index to determine the classification threshold for optimal balance performance of the model. The thresholds (cut-off) for clinical model, RS model, and integrated model were 0.393, 0.375, and 0.266 respectively. From the **Figure 5**, it can be observed that at their respective optimal threshold points, compared to clinical model, our integrated model exhibits an NRI of 0.244 for predicting positive patients and 0.062 for negative patients.

**Figure 6:**
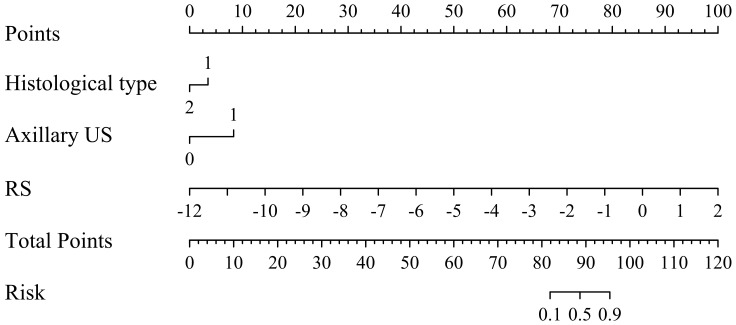
Nomogram for predicting the probability of axillary lymph node (ALN) metastasis risk in breast cancer patients ≥ 75 years.

**Figure 7:**
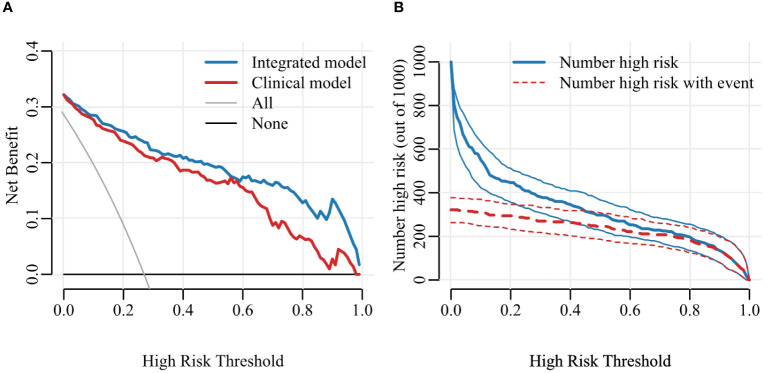
Decision curve **(A)** and Clinical Impact Curve (CIC) **(B)**. Decision curve analysis (DCA) allows threshold probability to vary to examine whether one model is superior to another at a certain range of threshold probability, with respect to “the net benefit”. CIC detects the predictive value, the red curve indicates the number of people classified as positive at each threshold probability and the blue curve is the number of true positives for each threshold probability.

To facilitate the clinical application, we have developed a nomogram model based on multiple logistic regression, as depicted in **Figure 6**. The decision curve and the Hosmer-Lemeshow test (with P = 0.613 for the training set and P = 0.979 for the validation set) demonstrate excellent predictive accuracy of the integrated model on both datasets. Furthermore, we have visually presented both the decision curve and clinical impact curve (as illustrated in **Figure 7**), thereby enhancing its applicability.

## Discussion

The objective of this study was to establish and validate a model based on DL and radiomics, which combines clinical features and ultrasound imaging, for predicting the ALN status in elderly breast cancer patients and providing reliable diagnostic information and treatment options for clinicians. Integrated model demonstrated excellent predictive performance on the training and validation sets, with AUC values of 0.937 and 0.906, respectively. These results indicate that integrated model can accurately predict the ALN status in elderly breast cancer patients and provide clinicians with reliable diagnostic information and treatment options.

Ultrasound has been widely used for preoperative assessment of ALN status in breast cancer. Although some studies have found that ultrasound-guided fine-needle aspiration or core needle biopsy, compared to SLNB, can save costs in clinically negative ALN patients (30), the accuracy of axillary ultrasound is still insufficient for predicting ALN status, with reported AUC values ranging from 0.585 to 0.719 (13). This indicates that axillary ultrasound still has limitations in predicting ALN status and cannot accurately predict LNM, which in turn may not support fine-needle aspiration or core needle biopsy. The accuracy of axillary ultrasound assessment in our study was also low, consistent with previous literature reports. Some studies have aimed to predict ALN status using clinical and imaging data, such as tumor grade, tumor size, lymphovascular invasion, and ultrasound spiculated margin (14–16, 31). However, relying solely on clinical and imaging predictors is not accurate enough, with AUC values ranging from 0.66 to 0.74 in previous studies. In comparison to the previous research, our study found that certain factors, such as histological classification and ultrasound lymph node assessment, have independent prognostic significance for ALN metastasis in elderly breast cancer patients. This differs to some extent from previous studies, which may be because previous research mainly focused on adult women and did not fully reflect the unique biological characteristics of elderly breast cancer patients. Important features, such as lymphovascular invasion, cannot be directly observed preoperatively, limiting their clinical significance in predicting ALN status. These differences underscore the importance of our study in further understanding ALN metastasis in elderly breast cancer patients.

In recent years, with the development of radiomics and DL, an increasing number of studies have applied these two approaches in clinical imaging research. For example, in studies using thyroid cancer ultrasound images, the application of radiomics allowed for the establishment of a model for predicting cervical LNM with high accuracy (AUC 0.914) (32). Similarly, in predicting ALN metastasis in breast cancer, radiomics and DL have also demonstrated high accuracy. For instance, Zheng et al. used DL combined with clinical features to build a model for predicting ALN metastasis based on ultrasound images of primary breast tumors, achieving a relatively high predictive accuracy (AUC 0.902) (33).

The novelty of this study resides in the integration of radiomics and DL techniques for constructing integrated model that comprehensively analyzes both clinical and ultrasound imaging features. When compared to conventional image evaluation methods, integrated model exhibits substantial enhancements in predicting ALN status, significantly increasing the AUC from 0.744 (as previously reported) to an impressive 0.906, thus demonstrating its exceptional accuracy and superiority. The enhanced predictive accuracy and reliability of integrated model stem from its comprehensive analysis and complementary interactions, which fully leverage ultrasound image information. Despite the inherent opacity limitations of DL models, our integrated approach incorporates traditional radiomics methods to compensate for this drawback by combining predictive results with imaging features. As a result, integrated model not only achieves superior predictive accuracy but also provides interpretable outcomes that can effectively aid doctors in understanding and applying them.

To further enhance the accuracy of prediction, we integrated clinical features with ultrasound imaging features to develop an integrated predictive model. The results demonstrated that the integrated model exhibited higher accuracy compared to models utilizing only clinical features or DL radiomics models. Notably, the negative predictive value even reached 0.893, indicating a high level of confidence in the model’s accuracy when predicting a negative ALN, approaching 90%. This indicates that when the prediction result of integrated model is no axillary LNM has a high accuracy, with the actual probability of no metastasis being close to 90%. The predictions of negative axillary lymph nodes made by integrated model can serve as a valuable reference for clinicians, enabling them to avoid unnecessary ALN surgeries and minimize the patient’s surgical risks and trauma. Instead, they can opt for a relatively conservative non-surgical treatment strategy such as radiation therapy, which offers improved disease control.

Despite our study’s achievements, there are still some limitations that need to be acknowledged. Firstly, we obtained data from various ultrasound devices and had them assessed by different physicians; this might introduce variations in equipment and operator performance that could potentially impact the results. Additionally, this study only includes ultrasound images which may underestimate the performance of DL without integrating other imaging modalities. Furthermore, our study was conducted solely at one center without external validation, thus restricting its generalizability. To enhance its credibility further, future research can utilize multicenter datasets for validation purposes while also comparing outcomes with other studies.

## Conclusion

The deep learning-and-radiomics-based ultrasound nomogram exhibits high accuracy and reliability in predicting the ALN status of breast cancer patients ≥ 75 years. It can serve as a valuable reference for clinicians to enhance the efficacy of individualized treatment strategies, helping patients to avoid unnecessary axillary lymph node surgery, thereby minimizing surgical risks and trauma while improving patients’ quality of life.

## Data availability statement

The original contributions presented in the study are included in the article/supplementary material. Further inquiries can be directed to the corresponding authors.

## Ethics statement

The studies involving humans were approved by Ethics Committee of Shanghai Cancer Institute, Fudan University. The studies were conducted in accordance with the local legislation and institutional requirements. The ethics committee/institutional review board waived the requirement of written informed consent for participation from the participants or the participants’ legal guardians/next of kin because As this study is retrospective in nature and does not disclose any identifiable information, informed consent was deemed unnecessary. Written informed consent was not obtained from the individual(s) for the publication of any potentially identifiable images or data included in this article because As this study is retrospective in nature and does not disclose any identifiable information, informed consent was deemed unnecessary.

## Author contributions

LQ: Data curation, Formal analysis, Methodology, Resources, Validation, Writing – original draft, Writing – review & editing. XL: Data curation, Formal analysis, Methodology, Resources, Validation, Writing – original draft, Writing – review & editing. SZ: Resources, Writing – review & editing. WZ: Resources, Writing – review & editing. KZ: Methodology, Writing – review & editing. HL: Validation, Writing – original draft, Writing – review & editing, Formal analysis, Methodology. CC: Validation, Writing – original draft, Writing – review & editing, Conceptualization, Resources. JL: Formal analysis, Methodology, Resources, Validation, Writing – original draft, Writing – review & editing.
